# A Systematic Review of the Clinical Impact of Implementing Artificial Intelligence in Upper Aerodigestive Tract Endoscopy

**DOI:** 10.1002/hed.28213

**Published:** 2025-06-18

**Authors:** Celine M. L. H. Wilmes, Arsen Goril BSc, Henri A. M. Marres, David J. Wellenstein, Guido B. van den Broek

**Affiliations:** ^1^ Department of Otorhinolaryngology and Head and Neck Surgery Radboud University Medical Center Nijmegen the Netherlands; ^2^ Department of Otorhinolaryngology and Head and Neck Surgery Rijnstate Hospital Arnhem the Netherlands; ^3^ Department of Information Management Radboud University Medical Center Nijmegen the Netherlands

**Keywords:** artificial intelligence, deep learning, endoscopy, machine learning, upper aerodigestive tract

## Abstract

**Background:**

Endoscopy is essential in upper aerodigestive tract (UADT) examination, particularly in the early detection of laryngopharyngeal lesions. However, UADT endoscopy remains operator‐dependent and lacks standardized quality metrics. Recent advancements in artificial intelligence (AI) have generated interest in applications within UADT endoscopy. This review evaluates the clinical impact of AI in UADT endoscopy.

**Methods:**

A literature review was conducted up to December 31, 2024. Studies were evaluated using the modified Quality Assessment of Diagnostic Accuracy Studies (QUADAS)‐2 tool.

**Results:**

Eighty‐three studies were included. Results indicate that AI in UADT endoscopy achieves diagnostic accuracy, sensitivity, and specificity rates comparable to experts, with optimal outcomes combined with human expertise. AI also demonstrated significantly faster inference times.

**Conclusions:**

This review highlights AI's potential to enhance clinical impact in UADT endoscopy, especially when combined with human expertise. However, the limited focus on real‐time clinical translation underscores the need for further research to enable effective integration into clinical practice.

## 1 Introduction

Upper aerodigestive tract (UADT) endoscopy is crucial in the examination of laryngopharyngeal function and pathology. Identifying pathological lesions and differentiating between benign and (pre)malignant lesions is essential. Notably, 6%–22% of premalignant UADT lesions will develop into malignancy, with transformation rates closely linked to the severity of the precancerous lesion [1]. Early and accurate distinction between potentially malignant and benign lesions is critical, as timely diagnosis and treatment significantly influence quality and survival outcomes [2]. Despite widespread utilization, this diagnostic process remains inherently operator‐dependent and lacks standardized, objective quality metrics [3]. The advent of high‐definition chip‐on‐tip endoscopy has transformed the practice of UADT endoscopy. These digital flexible endoscopes provide enhanced image resolution, facilitating more precise and accurate diagnoses of potentially malignant lesions at an earlier stage [4, 5, 6]. Additionally, this technology facilitates the storage and sharing of images, contributing to the development of large datasets, promoting collaborative care, improving follow‐up, and advancing telemedicine [7]. In parallel, there has been growing interest in incorporating artificial intelligence (AI) in UADT endoscopy to enhance diagnostic accuracy and efficiency.

Artificial intelligence, the development of computer systems capable of performing tasks that typically require human intelligence, has shown significant promise in medical imaging. In the context of UADT endoscopy, AI can potentially improve early lesion detection and reduce interoperator variability in endoscopy. Machine learning (ML) and deep learning (DL) are two subsets of AI that are particularly relevant in this context. Machine learning algorithms learn from large datasets and make predictions based on this data, requiring a form of feature extraction. Conventional ML techniques are limited in directly processing raw, unstructured data and typically rely on structured and processed datasets [8]. Deep learning, a more advanced subset of ML, utilizes neural networks composed of multiple layers to analyze raw, unstructured data, making it well‐suited for image analysis and pattern recognition [9, 10]. Supervised learning, the primary method of ML, involves training algorithms on labeled datasets, allowing them to generate binary outcomes based on specific features. Conversely, unsupervised learning relies on unannotated data to detect intrinsic qualities such as texture or color. Transfer learning (TL), which allows algorithms to apply knowledge gained from one task to a similar but different task, further expands the scope of ML and DL applications, reducing the time and resources required to train neural networks [9, 10, 11]. Transfer learning often leverages open‐access datasets, such as ImageNet, to enhance efficiency in image processing tasks [12]. Recent research demonstrates AI's capacity to improve diagnostic precision, reproducibility, and efficiency, particularly in radiology and pathology, where AI has been integrated into clinical practice in still images [13, 14].

In the realm of dynamic imaging, Maas et al. [15] reported a 40% increase in colorectal polyp detection using AI‐driven computer‐aided detection (CAD) systems, highlighting the potential of AI in real‐time endoscopy. These developments suggest that AI can improve UADT endoscopy by enhancing diagnostic precision for early laryngopharyngeal lesion detection and reducing interoperator variability.

Integrating AI in UADT endoscopy holds significant potential to improve diagnostic accuracy [16]. AI can provide critical support to laryngologists, reduce diagnostic errors, and enhance the education and training of residents. It is foreseen that AI‐assisted endoscopy may be utilized by general practitioners or speech therapists, enabling earlier detection of potentially malignant lesions. Ultimately, developing portable AI‐enabled endoscopes can facilitate screening and at‐home diagnosis, reducing the need for routine clinical visits.

Despite rapid advancements, the implementation of AI in UADT endoscopy remains in its early stages. This review is composed to evaluate the accuracy and efficiency of AI, guide its application, and centralize current research.

## 2 Methods

A literature review was conducted adhering to the PRISMA 2020 guidelines [17]. The protocol was registered in PROSPERO (CRD42023481426). The search strategy spanned eight databases (PubMed, Embase, Cochrane, Web of Science, Scopus, IEEE, ACM, and Reaxys) covering literature up to December 31, 2024. Publications were imported into EndNote, and duplicates were removed using Rayyan.ai [18]. Two independent reviewers (C.W. and A.G.) screened titles, abstracts, and full texts, resolving discrepancies through discussion. Study quality and risk of bias were assessed using a modified Quality Assessment of Diagnostic Accuracy Studies (QUADAS)‐2 tool (Table 1). Each criterion within the tool corresponds to a single point awarded for an affirmative response, with a maximum achievable score of 10. The total quality assessment (QA) scores for each study are summarized in the final column of Table 2. Studies were included if they (1) reported AI use in UADT endoscopy, (2) specified the AI algorithm and dataset utilized, and (3) assessed AI's clinical impact. Exclusion criteria were as follows: (1) nonstandard diagnostic methods (e.g., high‐speed endoscopy, contact‐endoscopy, micro laryngoscopy, gastroscopy), (2) nonhuman subjects, (3) non‐English publications, and (4) reviews or conference abstracts.

**TABLE 1:** Quality assessment questions based on the modified QUADAD‐2 tool.

Question	Score
Does the dataset consist of more than 500 unique frames?	0 or 1
Does the dataset consist of more than 1000 unique frames?	0 or 1
Is the test dataset separate from the training and validation dataset?	0 or 1
Is the dataset representative of the condition being assessed in the study?	0 or 1
Does the study involve one or more clinician(s) for annotations?	0 or 1
Is the algorithm compared to the clinician's performance?	0 or 1
Is the algorithm's performance compared to the clinician's training level?	0 or 1
Is the dataset multicentric?	0 or 1
Does the study fairly compare the outcomes of the AI methods to the existing methods?	0 or 1
Was the method described in sufficient detail to reproduce the presented results?	0 or 1
Total score (with a maximum of 10)	

**TABLE 2:** Summary of the main findings of the 83 included studies.

First author Year Country	Subsite of interest	Target health condition(s)	AI task	Best‐performing AI algorithm	Dataset Frame Patient	Open‐source dataset	Per‐formance metrics	Clinician	QA
Alrowais [19] 2023 Saudi Arabia	Larynx	Healthy, benign, (pre)malignant	Classification, detection	LCDC‐AOADL with InceptionV3, DBN, and AOA	1320 33	Zenodo Laryngeal [20]	Acc: 0.960 Pre: 0.921 Rec: 0.919 F1: 0.919	No	7
Alazwari [21] 2024 Saudi Arabia	Larynx	Healthy, benign, (pre)malignant	Classification, detection, segmentation	LCD‐CMDL	1320 33	Zenodo Laryngeal [20]	Acc: 0.970 Pre: 0.940 Rec: 0.941 F1: 0.940	No	5
Alzakari [22] 2024 Saudi Arabia	Larynx	Healthy, benign, (pre)malignant	Classification, detection	LCD‐DOAEL	1320 33	Zenodo Laryngeal [20]	Acc: 0.975 Pre: 0.950 Rec: 0.950 1: 0.949	No	7
Araujo [23] 2019 Portugal	Larynx	Healthy, benign, (pre)malignant	Classification, detection	LBPS + ResNet V2 with 101 layers layer B	1320 33	Zenodo Laryngeal [20]	Acc: 0.98 Pre: 0.98 Rec: 0.98 F1: 0.98	No	6
Ay [24] 2022 Turkey	Nasal cavity	Healthy, benign	Classification, detection	1CHL‐CNN	4289 766	ImageNet [12]	Acc: 0.977 Pre: 0.975 Rec: 0.975	No	7
Azam [25] 2024 Italy	Larynx	Malignant	Detection, segmentation	SegMENT‐Plus + SE, m‐ASPP, CBAM EfficiëntNetB5	4289 766	ImageNet [12]	Acc: 0.954 Pre: 0.779 Rec: 0.804	No	8
Azam [26] 2022 Italy	Larynx, pharynx, OC	Healthy, malignant	Classification, segmentation	SegMENT with Xception	901 219	ImageNet [12]	Acc: 0.973 Pre: 0.785 Rec: 0.951	No	7
Azam [27] 2022 Italy	Larynx	Healthy, malignant	Detection	YOLOv5s with YOLOv5m‐TTA	657 219	—	Pre: 0.664 Rec: 0.621	No	6
Baldini [28] 2024 Italy	Larynx	Healthy, benign, malignant	Classification, detection	ResNet‐50	5793 69	NBI‐InfFrames [29]	Pre: 0.97 Rec: 0.89 F1: 0.93	No	8
Bur [30] 2023 USA	Larynx	Benign, (pre)malignant	Classification, detection	ResNet‐50 with FPN, PAA	8172 147	COCO [31]	Acc: 0.881 Rec: 0.958	No	6
Chng [32] 2024 Singapore	Oro‐pharynx	Healthy, benign	Classification	EfificientNet B0	343 x	ImageNet [12]	Acc: 0.955 Pre: 1.0 Rec: 0.89 F1: 0.94	No	4
Cho [33] 2021 South Korea	Larynx	Healthy, benign, premalignant	Classification	EfficientNet‐B0	4106 4106	ImageNet [12]	Acc: 0.88 Pre: 0.89 Rec: 0.88 F1: 0.88	Yes	9
Cho [34] 2022 South Korea	Larynx	Healthy, benign, (pre)malignant	Classification, detection	VGG16 with OpenCV and Grad‐CAM	2216 2216	ImageNet [12]	Acc: 0.997 Pre: 0.993 Rec: 1.0	No	6
Dao [35] 2024 Vietnam	Larynx	Healthy, benign, malignant	Classification, detection	MEAL with CSP‐Darknet 53, YOLOv5, EfficientNetB0	1724 304	—	Acc: 0.951 Pre: 0.950 Rec: 0.948 F1: 0.951	No	7
Dao [36] 2024 Vietnam	Larynx	Healthy	Classification, detection	EfficientNetB1	4627 876	—	Acc: 0.987 Pre: 0.988 Rec: 0.986	No	6
Dunham [37] 2022 USA	Larynx	Healthy, benign, (pre)malignant	Classification	VGG16	19 353 x	—	Acc: 0.869 re: 0.874 Rec: 0.869	No	6
Fan [38] 2024 China	Larynx	Healthy, benign	Classification, segmentation	LFCF with DeepLabv3+ and IVCC	870 x	—	Acc: 0.972 Rec: 0.970	No	5
Fang [39] 2023 China	Larynx	Healthy, benign, (pre)malignant	Classification, detection	Faster R‐CNN + DropBlock	279 279	ImageNet [12]	Acc: 0.73 Rec: 0.73	Yes	5
Ganeshan [40] 2024 USA	Nasal cavity	Healthy	Classification, detection, segmentation	YOLOv8	2111 x	—	Acc: 0.915 Pre: 0.925 Rec: 0.938 F1: 0.931	No	5
Girdler [41] 2021 USA	Nasal cavity	Healthy, benign, premalignant	Classification, detection	ResNet‐152 with Grad‐CAM	297 297	ImageNet [12]	Acc: 0.742 Rec: 0.728 F1: 0.852	Yes	5
He [42] 2023 China	Naso‐ pharynx	Healthy, malignant	Detection	YOLOv8l	2429 690	ImageNet [12]	Pre: 0.825 Rec: 0.743 F1: 0.780	No	8
He [43] 2021 China	Hypo‐ pharynx	Benign, malignant	Classification, detection	InceptionV3	4591 4591	ImageNet [12]	Acc: 0.909 Rec: 0.901	Yes	8
Heo [44] 2022 South Korea	OC	Healthy, malignant	Classification, detection	DenseNet169	5576 x	ImageNet [12]	Acc: 0.830 Pre: 0.773 Rec: 0.793 F1: 0.777	Yes	9
Inaba [45] 2020 Japan	Larynx, pharynx	Healthy, malignant	Detection	RetinaNet with FPN	1200 185	ImageNet [12]	Acc: 0.973 Pre: 0.967 Rec: 0.955	No	6
Irem Turkmen [46] 2015 Turkey	Larynx	Benign	Classification, detection, segmentation	Vocal fold classifier based HOG	70 70	—	Pre: 0.81 Rec: 0.81	No	5
Joseph [47] 2024 India	Larynx	Healthy, benign, (pre)malignant	Classification	DenseNet 201 with LBP + STAT and RFE‐RF	1320 33	Zenodo Laryngeal [20]	Acc: 0.995 Pre: 0.995 Rec: 0.994 F1: 0.995	No	4
Joseph [48] 2024 India	Larynx	Healthy, benign, (pre)malignant	Classification	Modified SqueezeNet with XGBoost	1320 33	Zenodo Laryngeal [20]	Acc: 0.996 Pre: 1.00 Rec: 1.00 F1: 1.00	No	5
Kang [49] 2024 China	Larynx	Healthy, benign, malignant	Classification, detection	ILCDS with MobileNet V2	2023 610	ImageNet [12]	Acc: 0.960 Pre: 0.981 Rec: 0.983 F1: 0.982	No	8
Kang [50] 2024 China	Larynx	Healthy, benign, (pre)malignant	Classification, detection	ILCDS based on Swin‐Transformer	5768 1230	ImageNet [12]	Acc: 0.858 Pre: 0.831 Rec: 0.811 F1: 0.817	Yes	9
Kavak [51] 2024 Turkey	Larynx	Healthy, benign, premalignant	Classification	ADAM	91 159 433	—	Acc: 0.850 Pre: 0.856 Rec: 0.850 F1: 0.850	Yes	8
Kim [52] 2023 South Korea	Larynx	Healthy, benign, premalignant	Classification, detection	Yolo V4	2183 x	COCO [31]	Acc: 0.940 Pre: 0.883 Rec: 0.819 F1: 0.850	No	7
Kim [53] 2021 South Korea	Larynx	Healthy, benign, (pre)malignant	Detection	Mask RCNN with ResNet‐101	1224 x	COCO [31]	Acc: 0.62 Pre: 0.736 Rec: 0.802 F1: 0.767	No	6
Kono [54] 2021 Japan	Pharynx	Malignant	Classification, detection, segmentation	Mask RCNN	4559 276	—	Acc: 0.66 Pre: 0.55 Rec: 0.92	No	6
Kuo [55] 2021 China	Larynx	Healthy, benign, (pre)malignant	Classification, detection, segmentation	SVM with decision tree method	284 284	—	Acc: 0.933	Yes	4
Kuo [56] 2020 Taiwan	Larynx, pharynx	Malignant	Detection	RGB, L*a*b, YCbCr, CLAHE, Sobel, Otsu's, ACM, K‐means, FCM	359 359	—	Acc: 0.97	No	3
Kwon [57] 2022 South Korea	Larynx	Healthy, benign, (pre)malignant	Classification	Customized CNN model with CART algorithm, Grad‐CAM	36 630 471	—	Acc: 0.891 Pre: 0.886 Rec: 0.912 F1: 0.899	No	5
Kwon [58] 2024 South Korea	Nasal cavity	Healthy, benign, (pre)malignant	Classification, detection	Xception	474 680 5098	ImageNet [12]	Acc: 0.794 Rec: 0.794 F1: 0.790	Yes	9
Li [59] 2023 China	Larynx, pharynx	Healthy, benign, malignant	Classification, detection, segmentation	LPAIDS based on U‐Net	56 606 2382	—	Acc: 0.940 Pre: 0.950 Rec: 0.950	Yes	9
Li [60] 2023 China	Oro, hypo‐ pharynx	Benign, malignant	Classification, detection, segmentation	AI‐Adaboost consisting of LRPM‐HAM, RAM	600 x	—	Acc: 0.942	No	3
Li [61] 2018 South Korea	Naso‐pharynx	Benign, malignant	Classification, detection, segmentation	eNPM‐DM	63 903 7951	—	Acc: 0.887 Pre: 0.922 Rec: 0.913	Yes	8
Mamidi [62] 2024 USA	Larynx	Healthy, benign, (pre)malignant	Classification, detection, segmentation	ViT with YOLOv8	7513 182	—	Acc: 0.92 Pre: 0.91 Rec: 0.98 F1: 0.94	No	6
Moccia [63] 2018 Italy	Larynx	Malignant	Classification	BRISQUE, ΔVAR, TEN, ENTROPY, VAR, H, N_P, G_VAR, SVM + Gaussian	720 18	NBI‐InfFrames [29]	Pre: 0.803 Rec: 0.803 F1: 0.798	No	5
Moccia [64] 2017 Italy	Larynx	Healthy, benign, (pre)malignant	Classification	HLBPsPriu2 + Stat1 and SVM	1320 33	Zenodo Laryngeal [20]	Pre: 0.94 Rec: 0.93 F1: 0.92	No	6
Mohamed [65] 2023 Saudi Arabia	Larynx	Healthy, benign, (pre)malignant	Classification, detection	ALCAD‐DMODL technique based on EfficientNetB0	1320 33	Zenodo Laryngeal [20]	Acc: 0.972 Pre: 0.943 Rec: 0.943 F1: 0.943	No	7
Mohammed [66] 2018 Malaysia	Larynx	Healthy, benign, (pre)malignant	Classification, segmentation	Gabor filter and GLCM with ANN	381 x	—	Pre: 0.923 Rec: 0.939	No	3
Munirathinam [67] 2023 India	Larynx	Healthy, benign, (pre)malignant	Classification	InceptionV3 with SVM	1320 33	Zenodo Laryngeal [20]	Acc: 0.970 Pre: 0.972 Rec: 0.895 F1: 0.971	No	4
Nakajo [68] 2023 Japan	Larynx, pharynx	Healthy	Classification	Xception with Guided Grad‐CAM	6 492 586	—	Acc: 0.933 Pre: 0.934 Rec: 0.933 F1: 0.933	No	6
Paderno [69] 2023 Italy	Larynx, pharynx, OC	Malignant	Classification, detection, segmentation	Mask R‐CNN with RPN as backbone	1034 323	COCO [31]	Acc: 0.98 Pre: 0.94 Rec: 0.91 F1: 0.92	No	6
Paderno [70] 2021 Italy	OC, Oro‐pharynx	Malignant	Segmentation	FCNN ResNet	226 79	—	Acc: 0.853 Pre: 0.758 Rec: 0.754	No	3
Paderno [71] 2024 Italy	Oro‐pharynx	Malignant	Classification, detection, segmentation	DINOv2	327 38	—	Acc: 0.92 Pre: 0.93 Rec: 0.92 F1: 0.92	No	5
Parker [72] 2021 USA	Larynx	Benign	Segmentation	U‐Net	127 25	—	Pre: 0.367 Rec: 0.724	No	5
Patrini [73] 2020 Italy	Larynx	Malignant	Classification	VGG 16 with SVM	720 18	ImageNet [12], NBI‐InfFrames [29]	Pre: 0.936 Rec: 0.936 F1: 0.935	No	5
Rampinelli [74] 2024 Italy	Nasal cavity	Healthy, benign	Detection, segmentation	YOLOv8s‐seg	816 52	—	Pre: 0.910 Rec: 0.839	No	5
Ren [75] 2020 China	Larynx	Healthy, benign, (pre)malignant	Classification	ResNet‐101	24 667 9231	ImageNet [12]	Acc: 0.94 Rec: 0.94	Yes	7
Sampieri [76] 2024 Italy	Larynx	Benign, malignant	Classification, detection, segmentation	SegMENT‐Plus	4289 766	—	Acc: 0.973 Pre: 0.810 Rec: 0.900	Yes	9
Sobhi [77] 2021 Egypt	Larynx	Malignant	Classification, detection	BRISQUE, ΔDoM, Brenner, Tenegrad, Laplacian Gradient, Entropy, Energy, RF	720 18	NBI‐InfFrames [29]	Pre: 0.959 Rec: 0.958 F1: 0.958	No	5
Tai [78] 2024 South Korea	Naso‐pharynx	Healthy, benign, premalignant	Classification, detection, segmentation	InceptionResNetV2	9568 1442	ImageNet [12]	Acc: 0.823 Pre: 0.78 Rec: 0.90 F1: 0.84	Yes	9
Takiyama [79] 2023 Japan	Larynx, pharynx	Healthy	Classification	CNN based on GoogleNet	7335 1750	—	Acc: 0.974 Rec: 0.939	No	6
Tamashiro [80] 2020 Japan	Pharynx	Malignant	Detection	Single Shot MultiBox Detector	5403 35	—	Acc: 0.674 Pre: 0.607 Rec: 0.797	No	6
Tao [81] 2024 China	Larynx, pharynx	Foreign bodies (fish bones)	Detection	YOLO‐V5	3133 1406	COCO [31]	Acc: 0.812 Pre: 0.909 Rec: 0.818 F1: 0.87	Yes	9
Tie [82] 2024 China	Larynx	Benign, malignant	Classification, detection, segmentation	Deeplabv3‐resnet101	8248 487	—	Acc: 0.88 Pre: 0.882 Rec: 0.882	Yes	9
Tran [83] 2023 Vietnam	Larynx	Healthy, benign, (pre)malignant	Classification	Xception with Guided Grad‐CAM	4549 876	ImageNet [12]	Acc: 0.975 Pre: 0.939 Rec: 0.961 F1: 0.948	Yes	9
Tsung [84] 2022 Taiwan	Larynx	Benign, malignant	Classification, detection	EVC‐DD	1740 x	—	Acc: 0.994 Pre: 0.994 Rec: 0.994 F1: 0.994	No	7
Wang [85] 2024 Italy	Larynx	Benign, (pre)malignant	Classification, detection	LDA	11 144 210	Zenodo CE‐NBI [86]	Acc: 0.939 Pre: 0.953 Rec: 0.851 F1: 0.899	No	7
Wang [87] 2024 China	Naso‐pharynx	Healthy, benign, malignant	Classification, detection	YOLOv5l	17 608 2965	—	Acc: 0.948 Pre: 0.870 Rec: 0.942 F1: 0.904	Yes	9
Wang [88] 2024 China	Larynx	Healthy, benign, (pre)malignant	Classification	HDCNet, combination ResNet34 + ResNet50	3057 1950	Laryngo‐scope8 [89]	Acc: 0.753	No	7
Wang [90] 2022 China	Larynx	Benign, (pre)malignant	Classification, detection	Deeplabv3‐resnet101	5362 551	—	Acc: 0.850 Pre: 0.909 Rec: 0.784	Yes	9
Wellenstein [91] 2023 Netherlands	Larynx	Healthy, benign, (pre)malignant	Classification, detection	Yolov5s/ YOLOv5m	4754 649	Laryngo‐scope8 [89], COCO [31]	Pre: 0.700 Rec: 0.730 F1: 0.710	No	7
Xiong [92] 2019 China	Larynx	Healthy, benign, (pre)malignant	Classification, detection	Inception v3	29 812 2208	ImageNet [12]	Acc: 0.867 Rec: 0.731	Yes	10
Xiong [93] 2024 China	Larynx	(Pre)malignant	Classification, detection, segmentation	Mask R‐CNN + ResNet50 as backbone	6180 216	COCO [31]	Pre: 0.960 Rec: 0.975	No	5
Xu [94] 2022 China	Naso‐pharynx	Benign, malignant	Classification	S‐DCCN with CAM	4783 671	—	Acc: 0.957 Pre: 0.945 Rec: 0.970	No	5
Xu [95] 2023 China	Larynx	Benign, malignant	Classification, detection	DenseNet201	2254 428	ImageNet [12]	Acc: 0.863 Rec: 0.860	Yes	10
Yan [96] 2023 China	Larynx	Benign, malignant	Classification, detection	Faster R‐CNN	2179 2179	—	Acc: 0.781 Pre: 0.325 Rec: 0.742	No	7
Yao [97] 2024 USA	Larynx	Healthy, benign	Classification	ResNet 18	55 133 129	ImageNet [12]	Acc: 0.85 Pre: 0.85 Rec: 0.85 F1: 0.85	Yes	8
Yao [98] 2022 USA	Larynx	Healthy, benign	Classification	ResNet‐18	22 132 115	ImageNet [12]	Pre: 0.785 Rec: 0.830 F1: 0.805	No	6
Yin [89] 2021 China	Larynx	Healthy, benign, (pre)malignant	Classification	DenseNet‐121 and Faster R‐CNN	3057 1950	Laryngo‐scope8 [89]	Acc: 0.73	No	7
You [99] 2023 China	Larynx	Healthy, benign, (pre)malignant	Classification	GoogleNet	932 x	ImageNet [12]	Acc: 0.903 Pre: 0.953 Rec: 0.948	No	6
You [100] 2024 China	Larynx	Healthy, benign, (pre)malignant	Classification, detection	Very deep siamese network (DenseNet + Siamese net)	236 45	ImageNet [12]	Acc: 0.976 Rec: 0.981	No	6
Yue [101] 2024 China	Nasal cavity	Healthy, benign, malignant	Classification, detection	SwinT	39 340 13 958	—	Acc: 0.923 Rec: 0.964	No	8
Zhang [102] 2023 China	Larynx	Malignant	Classification	UMAP + Agglomerative clustering	720 18	NBI‐ InfFrames [29]	Pre: 0.953 Rec: 0.945 F1: 0.948	No	4
Zhao [103] 2022 China	Larynx	Healthy, benign, (pre)malignant	Classification	MobileNet‐V2	5122 x	ImageNet [12]	Acc: 0.802 F1: 0.784	Yes	8
Zhou [104] 2023 China	Larynx, pharynx	Benign, malignant	Detection, segmentation	PSA Net	15 198 300	ImageNet [12]	Acc: 0.963	No	7
Zhu [105] 2023 China	Larynx, pharynx, OC	Healthy	Classification	ILMA consisting of Inception‐ResNet‐v2 + SENet	20 000 6673	—	Acc: 0976 Pre: 0.977 Rec: 1.00	No	8

Abbreviations: Acc, accuracy; ACM, active contour method; ADAM, alternative adaptive boosting method; AI‐Adaboost, alternative adaptive boosting method; ALCAD‐DMODL, automated laryngeal cancer detection and classification using a dwarf mongoose optimization algorithm with deep learning; ANN, artificial neural network; AOA, aquila optimization algorithm; BRISQUE, blind/referenceless image spatial quality evaluator; CAM, class activation mapping; CART, classification and regression tree; CBAM, channel block attention module; CE‐NBI, contact endoscopy—narrow band imaging; CLAHE, contrast‐limited adaptive histogram equalization; CNN, convolutional neural network; COCO, common objects in context; DBN, deep belief network; DoM, deference of differences in grayscale values of a median‐filtered image; eNPM‐DM, endoscopic images‐based nasopharyngeal malignancy detection model; ENTROPY, image entropy; EVC‐DD, edge‐based vocal cord disease detecting system; F1, F1‐score; FCM, Fuzzy c‐means clustering algorithm; FCNN, fully convoluted neural networks; FPN, feature pyramid network; G_VAR, image intensity variance; GLCM, gray‐level co‐occurrence matrix; Grad‐CAM, gradient‐weighted class activation mapping; H, image histogram; HDCNet, hierarchical dynamic convolutional; HLBP, histogram of local binary patterns; HOG, histogram of oriented gradients; ILCDS, intelligent laryngeal cancer detection system; ILMA, intelligent laryngoscopy monitoring assistant; IVCC, inner vocal cord contour extraction; LBP, local binary pattern; LBPS, local binary pattern with first‐order statistics; LCDC‐AOADL, laryngeal cancer detection and classification using the aquila optimization algorithm with deep learning; LCD‐CMDL, laryngeal cancer detection using the chaotic metaheuristics integration with the deep learning; LCD‐DOAEL, laryngeal cancer diagnosis using the dandelion optimizer algorithm with ensemble learning; LDA, linear discriminant analysis; LFCF, local fine‐grained contour feature; LPAIDS, laryngopharyngeal artificial intelligence diagnostic system; LRPM‐HAM, local region proposal model with attentive temporal–spatial pathways; m‐ASPP, modified atrous spatial pyramid pooling; MEAL, multitask efficient transformer network for laryngoscopy; N_P, number of keypoints; NBI, narrow band imaging; OC, oral cavity; PAA, probabilistic anchor assignment; Pre, precision; RAM, recurrent attention model; RCNN, region based convolutional neural network; Rec, recall; RF, random forest; RFE‐RF, recursive feature elimination with random forest; RGB, red green blue; RPN, region proposal network; S‐DCNN, siamese deep convolutional neural network; SE, squeeze excitation; SENet, squeeze‐and‐excitation networks; STAT, single‐intensity‐based feature set; SVM, support vector machine; TEN, Sobel‐tenegrad focus evaluation function; TTA, test time augmentation; UMAP, uniform manifold approximation and projection; VAR, local variance of the luminance channel intensity; ViT, vision transformer; YOLO, you only look once.

This review evaluated the clinical impact of AI in UADT endoscopy, focusing on four areas:

1. AI characteristics (e.g., year of publication, country of origin, study type, AI subtype, open‐source dataset utilization, and anatomical subtype);
2. AI performance related to data volume, patient cohorts, and light source;
3. AI versus clinicians' performance in relation to experience level;
4. Clinicians' performance using AI.

Diagnostic performance is evaluated using several metrics: accuracy; precision (positive predictive value), reflecting the number of detected elements relevant; recall (sensitivity), representing the number of relevant elements detected; and the F1‐score, representing the harmonic mean of precision and recall.

$$\text{Accuracy}=\frac{\text{True Positive}\;\left(\mathrm{TP}\right)+\text{True Negative}\;\left(\mathrm{TN}\right)}{\text{True Positive}\;\left(\mathrm{TP}\right)+\text{True Negative}\;\left(\mathrm{TN}\right)+\text{False Positive}\;\left(\mathrm{FP}\right)+\text{False Negative}\;\left(\mathrm{FN}\right)}$$

$$\text{Precision}\;\left(P\right)=\frac{\mathrm{TP}}{\mathrm{TP}+\mathrm{FP}}$$

$$\text{Recall}\;\left(R\right)=\frac{\mathrm{TP}}{\mathrm{TP}+\mathrm{FN}}$$

$$F1-\text{score}=2*\frac{P*R}{P+R}$$

## 3 Results

**FIGURE 1:**
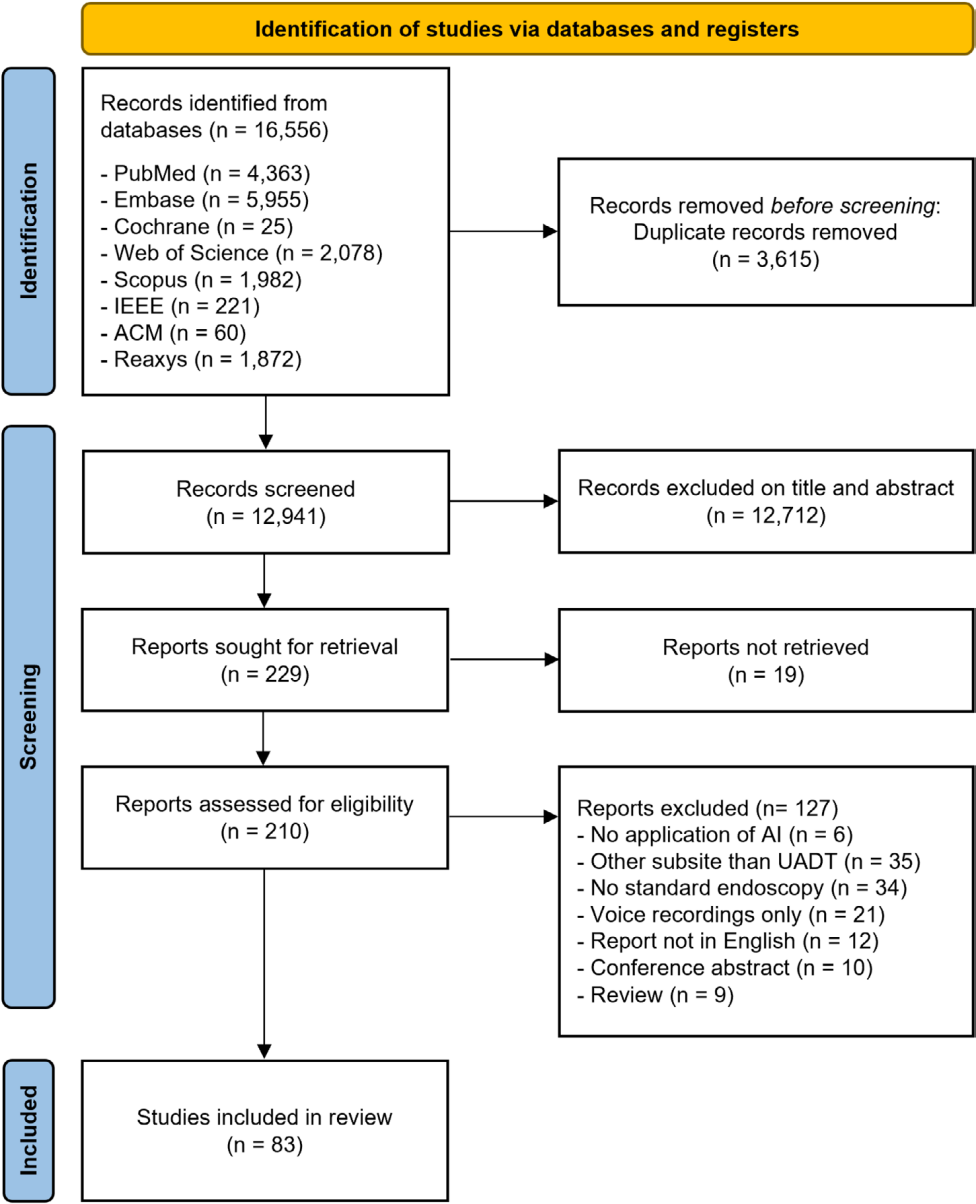
PRISMA flow diagram illustrating the systematic selection process of included studies [17]. [Color figure can be viewed at wileyonlinelibrary.com]

After duplicate removal, 12 941 unique records were identified. Following title and abstract screening, 12 712 were excluded for not meeting the inclusion criteria. Of 210 full texts assessed, 127 were excluded, resulting in 83 publications in the final review (Figure 1).

### 3.1 AI Characteristics

Table 2 summarizes the included studies, detailing the first author, year of publication, country of origin, anatomical subsite, target condition, AI task, and best‐performing AI algorithm utilized. Dataset characteristics (frame count, patient sample size, open‐source dataset utilization), performance metrics of the best‐performing algorithms, including comparisons to clinician performance, and the study QA are provided.

### 3.2 Year of Publication

**FIGURE 2:**
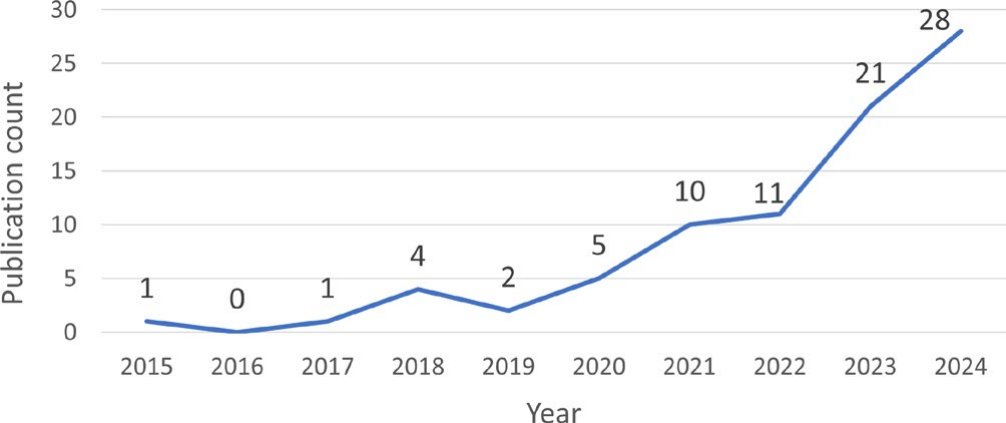
Overview of the number of publications per year. [Color figure can be viewed at wileyonlinelibrary.com]

Publication volume has risen sharply, with 49 of the 83 included studies published in the last 2 years (Figure 2). Before this recent increase, the average annual publication rate for AI in UADT endoscopy was around four.

### 3.3 Country of Origin

A comprehensive analysis of the global distribution of included publications indicates that China contributes the most, with 28 publications. Italy follows with 13 publications, predominantly published by a single research group [25, 26, 27, 28, 63, 64, 69, 70, 71, 73, 74, 76]. The geographic distribution of research outputs demonstrates a notable concentration in Western nations, reflecting the expansion of AI applications, particularly in economically developed regions.

### 3.4 Single‐ or Multicenter Studies and Prospective and Retrospective Data

Most of the included studies (78.3%) are single‐center studies, although the use of multicenter data for algorithm training has increased in recent years. Kwon et al. [58] built a large‐scale dataset of biopsy‐confirmed nasal cavity endoscopic images from 17 medical centers. Of the included studies, only four (4.8%) exclusively utilized prospective data, while two (2.4%) incorporated both prospective and retrospective data [61, 105]. Most studies (92.8%) relied solely on retrospective data.

### 3.5 AI Subtype

Over the past decade, the development of AI software in UADT endoscopy has undergone significant evaluation. Initially, the field of AI software in UADT endoscopy was predominantly characterized by the application of ML techniques; however, since 2018, there has been a transition toward DL methodologies, with most algorithms developed since 2021 based on DL approaches. More recently, an emerging trend toward integrating ML with DL techniques has emerged [47, 82, 90].

### 3.6 Open‐Source Dataset Utilization

Developing algorithms for UADT endoscopy necessitates large and diverse image‐based datasets that capture lesions under various conditions and perspectives (e.g., with the presence of saliva or blurred vision). Algorithms trained exclusively on self‐developed datasets encounter limitations in generalizability, prompting the adoption of techniques like data augmentation and the use of general‐domain open‐source datasets. A common approach uses pre‐existing general‐domain datasets to pretrain UADT endoscopy algorithms [12, 31]. One such example is ImageNet [12], a publicly available dataset comprising approximately 1.2 million images categorized into 1000 classes, including objects such as animals, vehicles, and buildings. During pretraining, algorithms learn fundamental image features such as edges, textures, and shapes, which are transferred to more complex tasks. This training approach mitigates the need to learn basic features from medical images, which are often limited in availability for medical applications.

**FIGURE 3:**
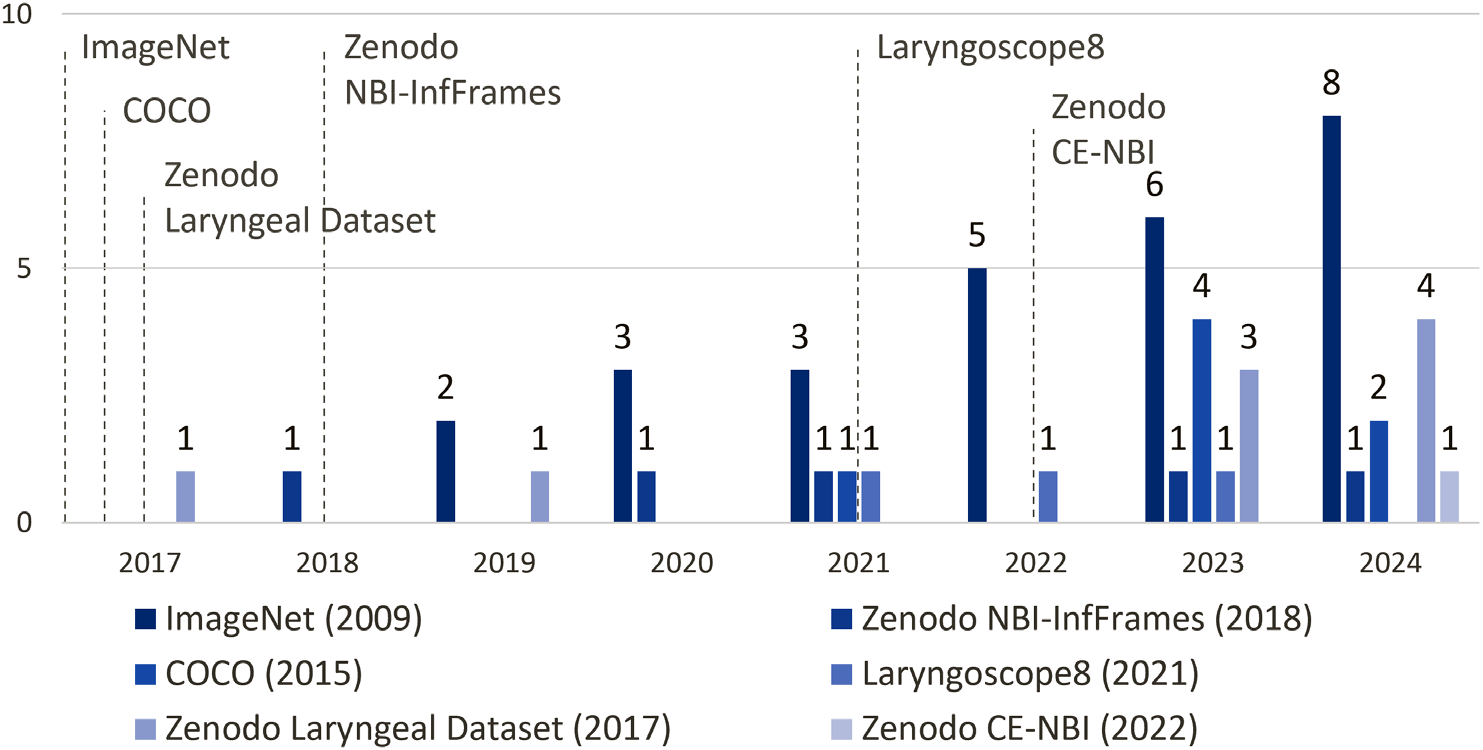
Bar chart depicting the annual utilization of open‐source datasets. [Color figure can be viewed at wileyonlinelibrary.com]

In addition to general‐domain datasets, specific in‐domain laryngeal image datasets, such as Laryngoscope8 [89], Zenodo Laryngeal Dataset [20], NBI‐InfFrames [29], and CE‐NBI [86], are increasingly used in the training of UADT endoscopy algorithms. Over recent years, several open‐source datasets have been established to support AI training and validation in this field, enhancing the accessibility and reproducibility of AI applications in UADT endoscopy [12, 20, 29, 31, 86, 89]. Figure 3 presents an overview of the open‐source datasets employed in the reviewed studies and their respective publication years.

### 3.7 Classification in Anatomical Subsite

AI applications in UADT endoscopy have predominantly focused on laryngeal subsites. Recently, there has been an increased emphasis on pharyngeal subsites, with Italian research groups leading studies on the oropharynx and oral cavity [26, 69, 70, 71]. In 2024, an increase in research targeting the nasal cavity was observed [40, 58, 74, 101].

### 3.8 AI Performance

Although specific algorithms are frequently used across studies, substantial differences in training methodologies, dataset selection, and open‐source and institution‐specific data integration complicate comparison. Most studies report accuracy, precision, recall, and F1‐score performance. Therefore, these metrics were selected to present performance outcomes.

### 3.9 AI Performance Related to Data Volume and Patient Cohorts

**FIGURE 4:**
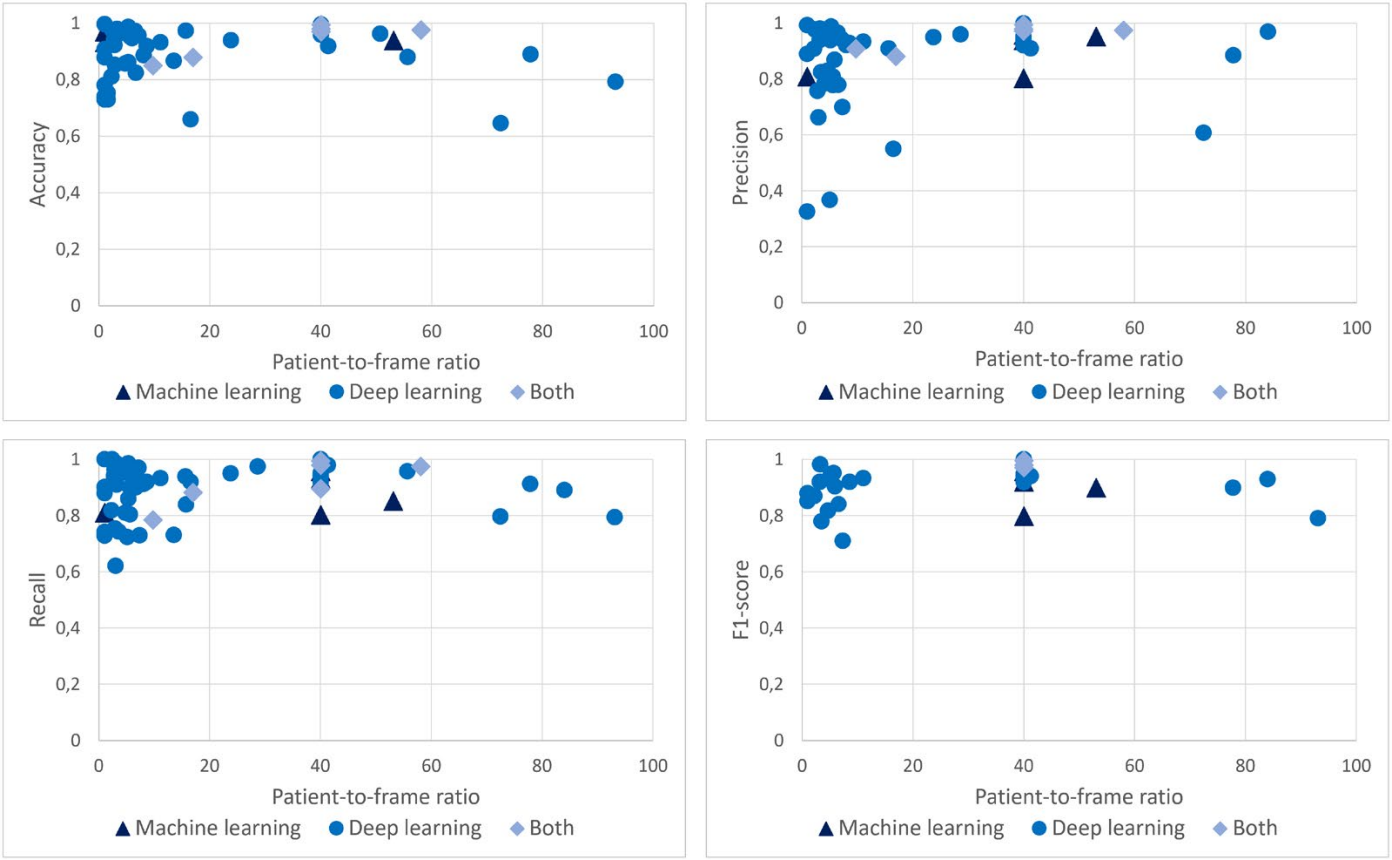
Comparison of accuracy, precision, recall, and F1‐score in relation to patient‐to‐frame ratio. [Color figure can be viewed at wileyonlinelibrary.com]

Table 2 provides a comprehensive overview of the algorithms utilized for detecting and classifying UADT lesions across the included studies. Figure 4 illustrates the performance metrics of the best‐performing algorithm from each study, contextualized by the patient‐to‐frame ratio—defined as the number of image frames acquired per patient. This ratio serves as an indicator of dataset diversity and potential overfitting risk [100]. As shown in Figure 4, algorithmic performance remains consistently high across varying patient‐to‐frame ratios, with no apparent decline at higher ratios. However, the predominance of lower patient‐to‐frame ratios among the included studies further supports the concern that overfitting is a prevalent issue in current research.

### 3.10 AI Performance Based on Light Source Utilization

**FIGURE 5:**
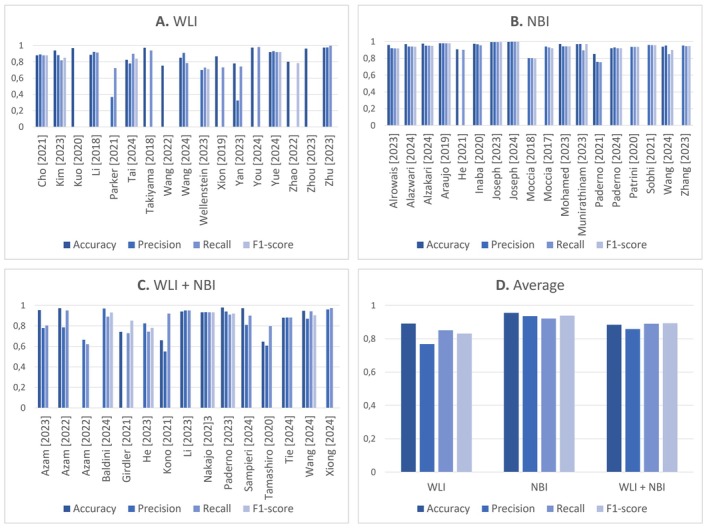
Overview of accuracy, precision, recall, and F1 score across studies, categorized by the type of light source utilized. [Color figure can be viewed at wileyonlinelibrary.com]

The reviewed studies utilized various light sources for imaging, including white light imaging (WLI), narrow‐band imaging (NBI), and combinations of both. Figure 5 compares key performance metrics—accuracy, precision, recall, and F1 scores—across studies employing these different light sources. The data consistently demonstrate that NBI outperforms WLI across all evaluated metrics—the combination of WLI and NBI results in lower performance across these metrics than NBI alone.

### 3.11 AI Versus Clinician Performance in Relation to Experience Level

**FIGURE 6:**
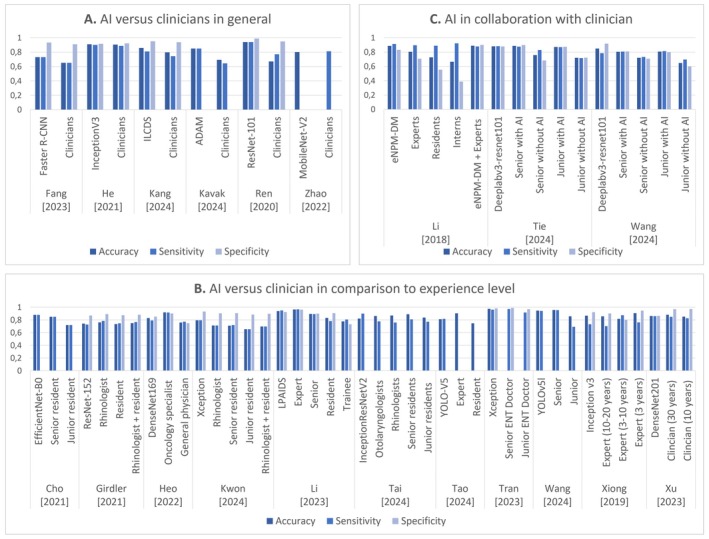
(A) Comparison of AI model performance with clinicians in general, (B) comparison of AI model performance in relation to the clinician's experience level, and (C) AI in collaboration with the clinician. [Color figure can be viewed at wileyonlinelibrary.com]

Figure 6 provides an overview of all included studies comparing AI performance with clinicians. However, not all studies explicitly compared AI performance to clinicians or reported comparable performance metrics, such as accuracy, sensitivity, or specificity [36, 55, 97, 105]. Dao et al. [36] demonstrated that AI could assist students in matching teacher performance without providing precise performance metrics. Kuo et al. [55] compared AI with clinician segmentation but only reported the average relative error. Yao et al. [97] presented confusion matrices for machine‐labeled and human‐labeled frames without directly comparing AI‐clinician performance. Finally, Zhu et al. [105] involved clinicians in reviewing images misclassified by the algorithm but did not include further comparative analysis.

### 3.12 Comparison of AI Algorithm Performance With Clinicians in General

Six of the 20 studies comparing AI performance with clinicians directly compared the best‐performing algorithms and clinicians in general [39, 43, 50, 51, 75, 103]. Figure 6A highlights the differences in accuracy, sensitivity, and specificity between the best‐performing algorithms and clinicians across these studies. Ren et al. [75] assessed their CNN algorithm compared to 12 clinicians, ranging from residents to attending and chief physicians. Their ResNet‐101 algorithm demonstrated superior performance to clinicians regarding accuracy, sensitivity, and specificity.

### 3.13 Comparison of AI Algorithm Performance in Relation to Clinicians' Experience Level

Eleven of the 20 studies that compared AI performance with clinicians stratified the results based on clinicians' experience levels [33, 41, 44, 58, 59, 78, 81, 83, 87, 92, 95]. Figure 6B presents an overview of these comparisons, focusing on accuracy, sensitivity, and specificity. In most studies, the algorithm exhibits performance comparable to that of the experienced clinician. Xu et al. [92] reported that the DenseNet201 algorithm achieved an area under the curve (AUC) of 0.926, which closely approximated the 0.927 AUC achieved by clinicians with over 30 years of experience and surpassed the 0.850 AUC of clinicians with over 10 years of experience. Tao et al. [81] evaluated detection speed by comparing the inference time of an AI algorithm—the time required for the algorithm to process new input data and generate predictions—with clinicians' performance. The algorithm demonstrated an inference time of 0.012 s per image, compared to 3.856 s for first‐year residents and 5.004 s for experts. Similarly, Sampieri et al. [76] achieved a dice similarity coefficient (DSC) and intersection over union (IoU) score of 0.89 for the SegMENT‐Plus algorithm, which was comparable to the scores of junior (0.88) and senior (0.91) residents.

### 3.14 Clinicians' Performance Using AI

Li et al. [61], Tie et al. [82], and Wang et al. [90] are the only publications in this review investigating the combined performance of clinicians and algorithms. Figure 6C compares the performance metrics for the algorithm, the clinician, and their combined approach. Li et al. [61] reported that integrating expert knowledge with the eNPM‐DM algorithm improved accuracy and specificity. However, the sensitivity of the combined approach remained comparable to that of the clinician working independently. A notable finding from these results is that combining a clinician and the AI algorithm yields optimal accuracy, sensitivity, and specificity performance.

## 4 Discussion

This systematic review provides the first comprehensive evaluation of the clinical impact of AI in UADT endoscopy. We analyzed the accuracy, sensitivity, and specificity of AI algorithms, clinicians, and their combination in detecting and classifying UADT lesions. Our findings demonstrate that AI performance is comparable to experienced clinicians, with optimal diagnostic outcomes in combination with human expertise. Furthermore, algorithms exhibited significantly faster inference times—the duration to process new data and generate predictions—emphasizing their potential for real‐time clinical applications. However, all included studies in this systematic review remain preclinical, with limited prospective clinical implementations. Most algorithms were trained on static images, primarily on laryngeal lesions, and often limited to a narrow spectrum of diagnoses. For clinical integration, future AI algorithms must demonstrate robust performance on dynamic video data across the entire laryngopharyngeal tract. Despite being in the early stages, the evidence strongly supports continued development for AI integration into clinical practice.

This review contributes to the growing literature on data sharing for algorithm training and validation [89, 91, 106, 107]. Patient privacy concerns and enormous storage demands of high‐resolution images complicate data sharing. While several open‐source datasets exist, most focus on general images rather than UADT lesions. Additionally, datasets containing UADT images are often small and based on a limited number of patients, resulting in data scarcity [31, 106]. Techniques like data augmentation help address this, but high‐quality UADT images remain limited. Notable datasets include Laryngoscope8, Zenodo's Laryngeal Dataset, NBI‐InfFrames, and CE‐NBI [20, 29, 86, 89]. Despite these resources, many studies rely on institution‐generated datasets supplemented with open‐source data. Collective data sharing improves AI performance and generalizability, facilitating accelerated development [108]. In addition, the review highlights a gap in research addressing the implementation of AI in clinical practice. Despite a recent increase in AI studies within UADT endoscopy, with 29 (34.9%) of the 83 included studies published in 2024, only 20 (24.1%) compared AI performance with clinicians, and just three (3.6%) evaluated the combined efficacy of AI and clinicians. Moreover, most studies were single‐center and retrospective, underscoring the need for multicenter datasets and prospective validation to enhance AI system generalizability.

Our findings emphasize several important considerations. To date, AI applications in UADT endoscopy have been mostly explored retrospectively. Algorithms are often trained and validated using data from a single center, with testing on datasets from the same institution. For instance, Li et al. [61] created the eNPM‐DM model, using DL for nasopharyngeal malignancy detection and biopsy guidance. The model was validated on a prospective test set of 1430 additional images, independent of the training, validation, and test sets. The eNPM‐DM model demonstrated comparable performance with clinicians, achieving an accuracy of 88.0% (95% CI 86.1%–89.6%), compared with 85.0% (95% CI 77.0%–84.0%) observed among clinicians.

No randomized controlled trials comparing the performance of AI and clinicians have been conducted in otolaryngology yet. In contrast, other specialties have made notable progress in integrating AI into endoscopic procedures to support clinical decision‐making. For example, Maas et al. [15] conducted a multicenter randomized trial comparing CAD colonoscopy with conventional colonoscopy in gastroenterology. A subset underwent tandem colonoscopy, alternating between CAD and conventional colonoscopy. The study showed that CAD improved adenoma detection rates and reduced adenoma miss rates without increasing non‐adenomatous lesion resections.

These findings underscore the need for prospective, real‐time AI applications in UADT endoscopy, where integration could enhance diagnostic accuracy and improve patient outcomes.

Data limitations arise from the sensitive nature of UADT lesion information. While images of laryngopharyngeal lesions are often pseudonymized, pathological confirmation is essential, posing significant challenges to data sharing and inter‐institutional collaboration. The general data protection regulation (GDPR) further complicates data exchange between research centers and commercial entities, hindering collaborative AI development and validation efforts in the Netherlands.

Additionally, most publications in this field originate from economically developed nations, likely due to the financial demands of algorithm development, high‐quality image equipment, and data storage. This limits data contributions from less developed countries, where AI‐driven UADT endoscopy could address shortages in medical professionals. Expanding access to algorithm development and validation is essential for reducing global health disparities in UADT diagnosis and treatment.

This review highlights several practical implications for advancing the implementation of AI in UADT endoscopy. The rapid advancements in the field emphasize the need to reassess data collection and sharing, prospective testing and validation of algorithms, and systematic AI integration into routine clinical practice.

First, collective data collection and sharing, supported by open‐source datasets, is crucial. Federated learning (FL), a distributed ML technique that operates with a central server and multiple clients, shows promise in healthcare by improving diagnostics, treatment planning, and disease prediction without sharing sensitive data. This collaboration through FL could enhance accuracy and personalization in healthcare outcomes [109]. However, current algorithm development practices—often based on single‐center datasets—need revision to improve robustness and generalizability. Training, validating, and testing algorithms on data from the same institution can lead to overly optimistic results, as these models may not generalize well to external datasets. This risk is further exacerbated when a lower patient‐to‐frame ratio—meaning a higher number of frames per patient—is present, as it is associated with better internal performance across evaluation metrics, regardless of whether ML or DL methods are used. While this might suggest improved accuracy, it often reflects overfitting, where models capture patient‐specific features rather than generalizable patterns. Testing on independent datasets is therefore essential to ensure the development of universally applicable, accurate, and clinically meaningful algorithms across multiple centers, minimizing overfitting and enhancing real‐world applicability [100].

Second, the results in this systematic review suggest that NBI use is associated with improved accuracy, sensitivity, and specificity performance. Interestingly, combining WLI and NBI results in lower accuracy, precision, recall, and F1‐score performance. This suggests that NBI may offer distinct advantages in detecting and classifying UADT lesions, possibly due to its enhanced ability to highlight mucosal structures and blood vessels, which are critical for accurate lesion identification [110]. Therefore, NBI holds considerable potential for future clinical applications in UADT endoscopy. However, it is important to note that many studies rely on simplified, publicly available NBI datasets, which could introduce bias due to their limited scope and homogeneity [29, 86]. Furthermore, very few studies directly compare performance on the same patient cohort using both NBI and WLI, making it difficult to draw definitive conclusions about the relative efficacy of these light sources under identical conditions. This lack of controlled comparison highlights a gap in the existing literature, suggesting the need for future studies that directly contrast the performance of these imaging modalities within the same patient populations.

Lastly, prioritizing multi‐institutional models is essential for broader clinical impact, improving diagnostic accuracy, timely treatments, and patient outcomes.

This review acknowledges several limitations. First, AI implementation in UADT endoscopy is still in its nascent stages. Most early studies, dating from 2015, focused on ML, with DL only emerging in 2018. While DL adoption has grown, the limited availability of large datasets has hindered AI development in UADT endoscopy, preventing progress in other fields like gastroenterology.

Second, although this review covers 83 studies, this number is insufficient for robust categorization, such as ML versus DL. Moreover, the heterogeneity of algorithms complicates direct comparisons, as variations in training, validation, and datasets lead to inconsistent outcomes.

Furthermore, many studies in the reviewed literature rely on static images for training algorithms, which limits their real‐time clinical applicability. Since endoscopic procedures involve dynamic, real‐time changes, training on video datasets is essential to ensure algorithms can operate effectively in clinical settings.

In addition, all the studies reviewed are preclinical, with no real‐world clinical implementations attempted thus far. As such, comparisons with physician performance lack clinical relevance, as they do not account for real‐world complexities. Future research should focus on clinical trials to assess the practical efficacy of these AI systems compared to physician performance under actual clinical conditions.

Lastly, most studies focused on healthy and malignant lesions, with few addressing benign and premalignant lesions. Larger and more diverse datasets are crucial for training algorithms on benign and (pre)malignant lesions, improving early detection of premalignant conditions, potentially facilitating timely intervention, and enhancing patient survival outcomes.

Despite these limitations, this systematic review provides compelling evidence for AI's potential in UADT endoscopy. AI's capacity to improve early detection and treatment of UADT lesions represents a significant advancement for the future of endoscopic practice.

In conclusion, this systematic review emphasizes the potential of AI in UADT endoscopy, demonstrating its ability to achieve high accuracy, sensitivity, and specificity in lesion detection and classification. The performance of several AI algorithms is comparable to or exceeds that of experienced clinicians, with optimal diagnostic outcomes observed when AI is integrated with human expertise. Notably, AI algorithms have demonstrated superior lesion detection rates, underscoring their potential to enhance real‐time detection and classification of lesions, facilitating more timely and accurate diagnosis.

However, while these advancements are promising, further research is essential to establish the clinical utility of AI in this field. Future efforts should focus on the development of standardized, evidence‐based protocols, as well as the implementation of robust clinical trials and prospective clinical validation studies. These endeavors will be crucial in ensuring the effective integration of AI technologies into routine clinical practice, ultimately advancing the diagnostic capabilities and efficiency of UADT endoscopy.

## Supporting information


Appendix A1.

## Data Availability

Data sharing not applicable to this article as no datasets were generated or analysed during the current study.
