# Supplementary material for: A Systematic Review of the Clinical Impact of Implementing Artificial Intelligence in Upper Aerodigestive Tract Endoscopy
Source: Head Neck. 2025 Jun 18;47(11):2998–3018. doi: 10.1002/hed.28213 (PMC12541685; doi:10.1002/hed.28213)
Supplement: Supplementary file 1 — Appendix A1. [file HED-47-2998-s001.docx]

# **Appendix A**

## Literature search strategy

The following databases were searched:
PubMed, Embase, Cochrane, Web of Science, Scopus, IEEE, ACM, and Reaxys

### PubMed

Date 21-11-2024
"( Artificial intelligence[mesh:noexp] OR ""Algorithms""[Mesh:NoExp] OR ""Machine Learning""[Mesh] OR ""Neural Networks, Computer""[Mesh] OR Support vector machine[mesh] OR Discriminant analysis[mesh] OR Artificial intelligence[tiab] OR Machine learning[tiab] OR Algorithm*[tiab] OR Computer neural network*[tiab] OR deep neural network*[tiab] OR Support vector machine*[tiab] OR Discriminant analys*[tiab] OR Deep learning*[tiab] OR Supervised learning*[tiab] OR AI[tiab] OR Artificial neural network*[tiab] OR Convolutional neural network*[tiab] OR CNN[tiab])",,,"""artificial intelligence""[MeSH Terms:noexp] OR ""Algorithms""[MeSH Terms:noexp] OR ""Machine Learning""[MeSH Terms] OR ""neural networks, computer""[MeSH Terms] OR ""support vector machine""[MeSH Terms] OR ""discriminant analysis""[MeSH Terms] OR ""artificial intelligence""[Title/Abstract] OR ""Machine Learning""[Title/Abstract] OR ""algorithm*""[Title/Abstract] OR ""computer neural network*""[Title/Abstract] OR ""deep neural network*""[Title/Abstract] OR ""support vector machine*""[Title/Abstract] OR ""discriminant analys*""[Title/Abstract] OR ""deep learning*""[Title/Abstract] OR ""supervised learning*""[Title/Abstract] OR ""AI""[Title/Abstract] OR ""artificial neural network*""[Title/Abstract] OR ""convolutional neural network*""[Title/Abstract] OR ""CNN""[Title/Abstract]","752,785",08:03:29

Date 31-12-2024
"((""Laryngoscopy""[Mesh] OR ""Laryngeal Diseases""[Mesh] OR ""Pharyngeal diseases""[Mesh] OR Videolaryngoscop*[tiab] OR laryn*[tiab] OR pharyn*[tiab] OR hypopharyn*[tiab] OR nasopharyn*[tiab] OR oropharyn*[tiab] OR vocal fold*[tiab] OR vocal cord*[tiab] OR glottis[tiab] OR glottic[tiab])) AND ((Artificial intelligence[mesh:noexp] OR ""Algorithms""[Mesh:NoExp] OR ""Machine Learning""[Mesh] OR ""Neural Networks, Computer""[Mesh] OR Support vector machine[mesh] OR Discriminant analysis[mesh] OR Artificial intelligence[tiab] OR Machine learning[tiab] OR Algorithm*[tiab] OR Computer neural network*[tiab] OR deep neural network*[tiab] OR Support vector machine*[tiab] OR Discriminant analys*[tiab] OR Deep learning*[tiab] OR Supervised learning*[tiab] OR AI[tiab] OR Artificial neural network*[tiab] OR Convolutional neural network*[tiab] OR CNN[tiab]))",,from 2023/11/21 - 2024/6/6,"((""Laryngoscopy""[MeSH Terms] OR ""Laryngeal Diseases""[MeSH Terms] OR ""Pharyngeal diseases""[MeSH Terms] OR ""videolaryngoscop*""[Title/Abstract] OR ""laryn*""[Title/Abstract] OR ""pharyn*""[Title/Abstract] OR ""hypopharyn*""[Title/Abstract] OR ""nasopharyn*""[Title/Abstract] OR ""oropharyn*""[Title/Abstract] OR ""vocal fold*""[Title/Abstract] OR ""vocal cord*""[Title/Abstract] OR ""glottis""[Title/Abstract] OR ""glottic""[Title/Abstract]) AND (""artificial intelligence""[MeSH Terms:noexp] OR ""Algorithms""[MeSH Terms:noexp] OR ""Machine Learning""[MeSH Terms] OR ""neural networks, computer""[MeSH Terms] OR ""support vector machine""[MeSH Terms] OR ""discriminant analysis""[MeSH Terms] OR ""artificial intelligence""[Title/Abstract] OR ""Machine Learning""[Title/Abstract] OR ""algorithm*""[Title/Abstract] OR ""computer neural network*""[Title/Abstract] OR ""deep neural network*""[Title/Abstract] OR ""support vector machine*""[Title/Abstract] OR ""discriminant analys*""[Title/Abstract] OR ""deep learning*""[Title/Abstract] OR ""supervised learning*""[Title/Abstract] OR ""AI""[Title/Abstract] OR ""artificial neural network*""[Title/Abstract] OR ""convolutional neural network*""[Title/Abstract] OR ""CNN""[Title/Abstract])) AND (2023/11/21:2024/6/6[pdat])",316,09:47:03

### Embase

Date run: 1974 to 2024 December 31

1 exp Laryngoscopy/ or exp larynx disorder/ or exp Pharynx disease/ or Videolaryngoscop*.ti,ab,kf. or laryn*.ti,ab,kf. or pharyn*.ti,ab,kf. or hypopharyn*.ti,ab,kf. or nasopharyn*.ti,ab,kf. or oropharyn*.ti,ab,kf. or vocal fold*.ti,ab,kf. or vocal cord*.ti,ab,kf. or glottis.ti,ab,kf. or glottic.ti,ab,kf. 372376

2 exp Artificial intelligence/ or exp Algorithm/ or exp Machine Learning/ or deep learning/ or Discriminant analysis/ or Artificial intelligence.ti,ab,kf. or Machine learning.ti,ab,kf. or Algorithm*.ti,ab,kf. or Computer neural network*.ti,ab,kf. or deep neural network*.ti,ab,kf. or Support vector machine*.ti,ab,kf. or Discriminant analys*.ti,ab,kf. or Deep learning*.ti,ab,kf. or Supervised learning*.ti,ab,kf. or AI.ti,ab,kf. or Artificial neural network*.ti,ab,kf. or Convolutional neural network*.ti,ab,kf. or CNN.ti,ab,kf. 1192521

3 1 and 2 8017

4 3 and 2 32: 2024.(sa_year).

### Cochrane

Date Run: 21-11-2023 23:01:41

ID Search Hits

#1 ([mh Laryngoscopy] OR [mh "Laryngeal Diseases"] OR [mh "Pharyngeal diseases"] OR Videolaryngoscop*:ti,ab,kw OR laryn*:ti,ab,kw OR pharyn*:ti,ab,kw OR hypopharyn*:ti,ab,kw OR nasopharyn*:ti,ab,kw OR oropharyn*:ti,ab,kw OR ("vocal" NEXT fold*):ti,ab,kw OR ("vocal" NEXT cord*):ti,ab,kw OR glottis:ti,ab,kw OR glottic:ti,ab,kw) 30649

#2 ([mh ^"Artificial intelligence"] OR [mh ^Algorithms] OR [mh "Machine Learning"] OR [mh "Neural Networks, Computer"] OR [mh "Support vector machine"] OR [mh "Discriminant analysis"] OR "Artificial intelligence":ti,ab,kw OR "Machine learning":ti,ab,kw OR Algorithm*:ti,ab,kw OR ("Computer neural" NEXT network*):ti,ab,kw OR ("deep neural" NEXT network*):ti,ab,kw OR ("Support vector" NEXT machine*):ti,ab,kw OR ("Discriminant" NEXT analys*):ti,ab,kw OR ("Deep" NEXT learning*):ti,ab,kw OR ("Supervised" NEXT learning*):ti,ab,kw OR AI:ti,ab,kw OR ("Artificial neural" NEXT network*):ti,ab,kw OR ("Convolutional neural" NEXT network*):ti,ab,kw OR CNN:ti,ab,kw) 26473

#3 #1 AND #2 339

Date Run: 31-12-2024 12:26:00

#1 ([mh Laryngoscopy] OR [mh "Laryngeal Diseases"] OR [mh "Pharyngeal diseases"] OR Videolaryngoscop*:ti,ab,kw OR laryn*:ti,ab,kw OR pharyn*:ti,ab,kw OR hypopharyn*:ti,ab,kw OR nasopharyn*:ti,ab,kw OR oropharyn*:ti,ab,kw OR ("vocal" NEXT fold*):ti,ab,kw OR ("vocal" NEXT cord*):ti,ab,kw OR glottis:ti,ab,kw OR glottic:ti,ab,kw) 32419

#2 ([mh ^"Artificial intelligence"] OR [mh ^Algorithms] OR [mh "Machine Learning"] OR [mh "Neural Networks, Computer"] OR [mh "Support vector machine"] OR [mh "Discriminant analysis"] OR "Artificial intelligence":ti,ab,kw OR "Machine learning":ti,ab,kw OR Algorithm*:ti,ab,kw OR ("Computer neural" NEXT network*):ti,ab,kw OR ("deep neural" NEXT network*):ti,ab,kw OR ("Support vector" NEXT machine*):ti,ab,kw OR ("Discriminant" NEXT analys*):ti,ab,kw OR ("Deep" NEXT learning*):ti,ab,kw OR ("Supervised" NEXT learning*):ti,ab,kw OR AI:ti,ab,kw OR ("Artificial neural" NEXT network*):ti,ab,kw OR ("Convolutional neural" NEXT network*):ti,ab,kw OR CNN:ti,ab,kw) 28576

#3 #1 AND #2 358

### Web of Science

Date run: Tue Nov 21 2023 15:08:55

1: (TS=(((Videolaryngoscop* OR laryn* OR pharyn* OR hypopharyn* OR nasopharyn* OR oropharyn* OR "vocal fold*" OR "vocal cord*" OR glottis OR glottic))) AND TS=(("Artificial intelligence" OR "Machine Learning" OR Algorithm* OR "Computer neural network*" OR "deep neural network*" OR "Support vector machine*" OR "Discriminant analys*" OR "Deep learning*" OR "Supervised learning*" OR AI OR "Artificial neural network*" OR "Convolutional neural network*" OR CNN)))
Results: 3956

### Scopus


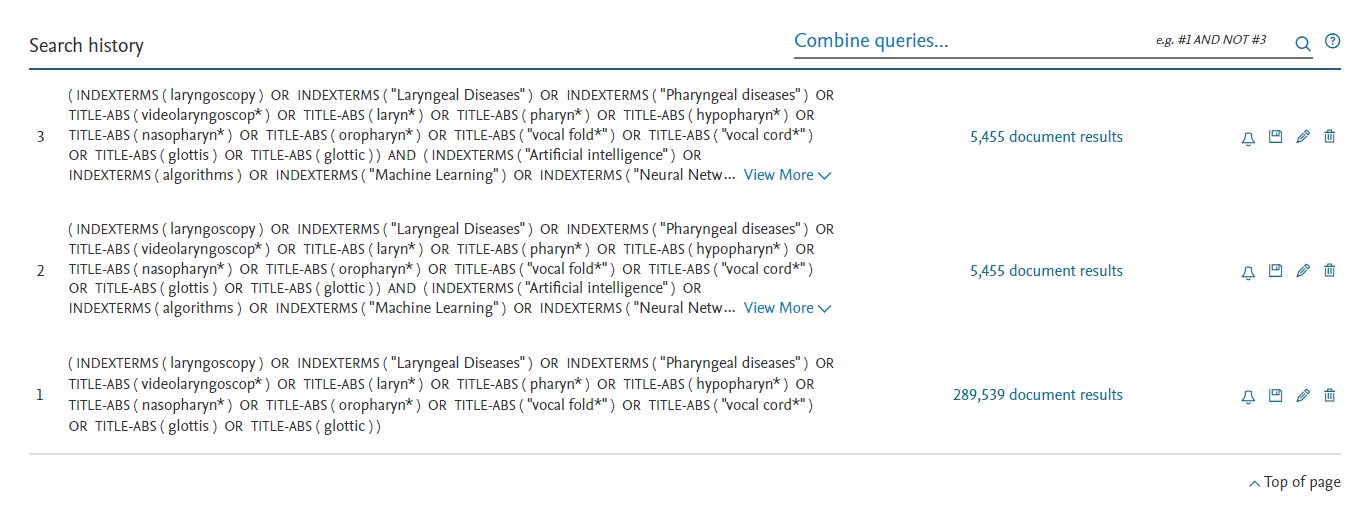
Date run: 21-11-2023 15:25u
Date run: 31-12-2023 15:57u


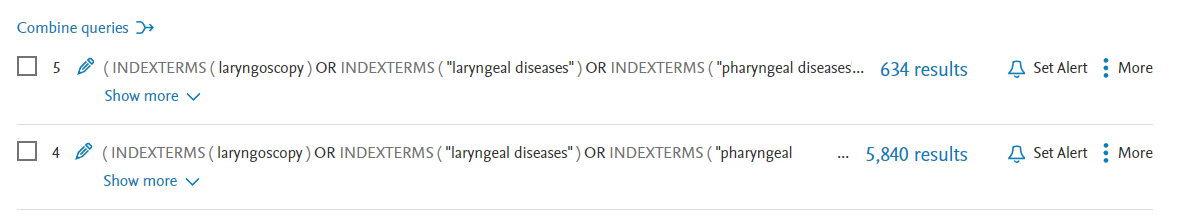


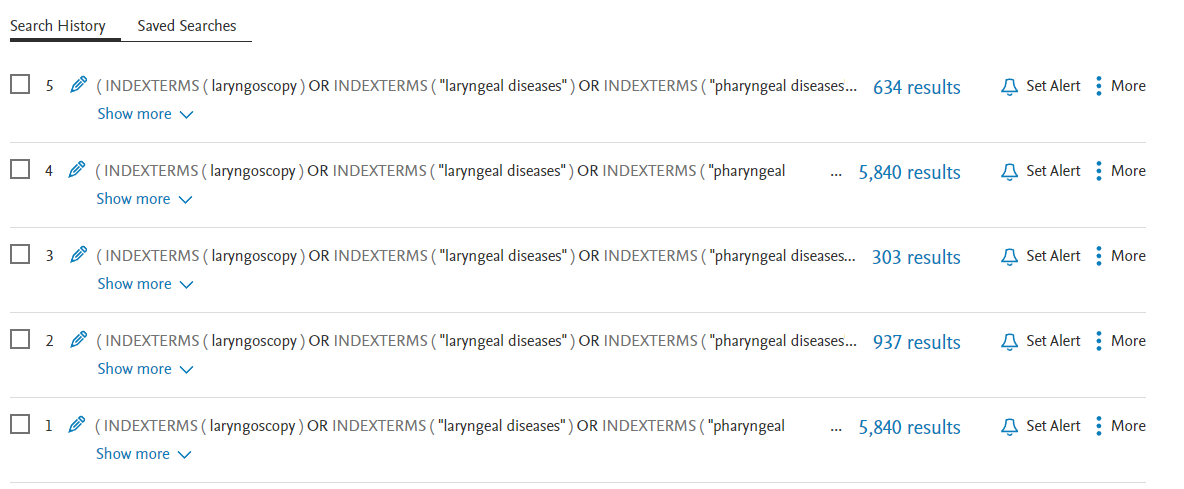


### IEEE

Date run: 21-11-2024

"(All Metadata:Videolaryngoscopy OR All Metadata:Videolaryngoscopic OR All Metadata:Videolaryngoscopies OR All Metadata:larynx OR All Metadata:laryngeal OR All Metadata:pharynx OR All Metadata:pharyngeal OR All Metadata:hypopharynx OR All Metadata:hypopharyngeal OR All Metadata:nasopharynx OR All Metadata:nasopharyngeal OR All Metadata:oropharynx OR All Metadata:oropharyngeal OR All Metadata:vocal fold OR All Metadata:vocal folds OR All Metadata:vocal cord OR All Metadata:vocal cords OR All Metadata:glottis OR All Metadata:glottic) AND (All Metadata:Artificial intelligence OR All Metadata:Machine Learning OR All Metadata:Algorithm* OR All Metadata:Computer neural network* OR All Metadata:deep neural network* OR All Metadata:Support vector machine* OR All Metadata:Discriminant analys* OR All Metadata:Deep learning* OR All Metadata:Supervised learning* OR All Metadata:AI OR All Metadata:Artificial neural network* OR All Metadata:Convolutional neural network* OR All Metadata:CNN)","",578,"November 21, 2023"

Date run: 31-12-2024

"(All Metadata:Videolaryngoscopy OR All Metadata:Videolaryngoscopic OR All Metadata:Videolaryngoscopies OR All Metadata:larynx OR All Metadata:laryngeal OR All Metadata:pharynx OR All Metadata:pharyngeal OR All Metadata:hypopharynx OR All Metadata:hypopharyngeal OR All Metadata:nasopharynx OR All Metadata:nasopharyngeal OR All Metadata:oropharynx OR All Metadata:oropharyngeal OR All Metadata:vocal fold OR All Metadata:vocal folds OR All Metadata:vocal cord OR All Metadata:vocal cords OR All Metadata:glottis OR All Metadata:glottic) AND (All Metadata:Artificial intelligence OR All Metadata:Machine Learning OR All Metadata:Algorithm* OR All Metadata:Computer neural network* OR All Metadata:deep neural network* OR All Metadata:Support vector machine* OR All Metadata:Discriminant analys* OR All Metadata:Deep learning* OR All Metadata:Supervised learning* OR All Metadata:AI OR All Metadata:Artificial neural network* OR All Metadata:Convolutional neural network* OR All Metadata:CNN)","Search Latest Date: 11/21/2023-06/06/2024",86,"June 6, 2024"

### ACM

Date run: 21-11-2023

("Videolaryngoscopy" OR "Videolaryngoscopic" OR "Videolaryngoscopies" OR "larynx" OR "laryngeal" OR "pharynx" OR "pharyngeal" OR "hypopharynx" OR "hypopharyngeal" OR "nasopharynx" OR "nasopharyngeal" OR "oropharynx" OR "oropharyngeal" OR "vocal fold" OR "vocal folds" OR "vocal cord" OR "vocal cords" OR "glottis" OR "glottic")

AND

("Artificial intelligence" OR "Machine Learning" OR Algorithm* OR "Computer neural network*" OR "deep neural network*" OR "Support vector machine*" OR "Discriminant analys*" OR "Deep learning*" OR "Supervised learning*" OR AI OR "Artificial neural network*" OR "Convolutional neural network*" OR CNN)

Date run: 31-12-2024

("Videolaryngoscopy" OR "Videolaryngoscopic" OR "Videolaryngoscopies" OR "larynx" OR "laryngeal" OR "pharynx" OR "pharyngeal" OR "hypopharynx" OR "hypopharyngeal" OR "nasopharynx" OR "nasopharyngeal" OR "oropharynx" OR "oropharyngeal" OR "vocal fold" OR "vocal folds" OR "vocal cord" OR "vocal cords" OR "glottis" OR "glottic")

AND

("Artificial intelligence" OR "Machine Learning" OR Algorithm* OR "Computer neural network*" OR "deep neural network*" OR "Support vector machine*" OR "Discriminant analys*" OR "Deep learning*" OR "Supervised learning*" OR AI OR "Artificial neural network*" OR "Convolutional neural network*" OR CNN)

Reaxys
Date run: 21-11-2024

"Videolaryngoscopy" OR "Videolaryngoscopic" OR "Videolaryngoscopies" OR "larynx" OR "laryngeal" OR "pharynx" OR "pharyngeal" OR "hypopharynx" OR "hypopharyngeal" OR "nasopharynx" OR "nasopharyngeal" OR "oropharynx" OR "oropharyngeal" OR "vocal fold" OR "vocal folds" OR "vocal cord" OR "vocal cords" OR "glottis" OR "glottic"

AND

"Artificial intelligence" OR "Machine Learning" OR Algorithm* OR "Computer neural network*" OR "deep neural network*" OR "Support vector machine*" OR "Discriminant analys*" OR "Deep learning*" OR "Supervised learning*" OR AI OR "Artificial neural network*" OR "Convolutional neural network*" OR CNN

Date run: 31-12-2024

"Videolaryngoscopy" OR "Videolaryngoscopic" OR "Videolaryngoscopies" OR "larynx" OR "laryngeal" OR "pharynx" OR "pharyngeal" OR "hypopharynx" OR "hypopharyngeal" OR "nasopharynx" OR "nasopharyngeal" OR "oropharynx" OR "oropharyngeal" OR "vocal fold" OR "vocal folds" OR "vocal cord" OR "vocal cords" OR "glottis" OR "glottic"

AND

"Artificial intelligence" OR "Machine Learning" OR Algorithm* OR "Computer neural network*" OR "deep neural network*" OR "Support vector machine*" OR "Discriminant analys*" OR "Deep learning*" OR "Supervised learning*" OR AI OR "Artificial neural network*" OR "Convolutional neural network*" OR CNN
